# Letter on “Early prediction of noninvasive ventilation failure in COPD patients: derivation, internal validation, and external validation of a simple risk score”

**DOI:** 10.1186/s13613-019-0613-9

**Published:** 2019-12-18

**Authors:** Emna Ennouri, Khaoula Meddeb, Radhouane Toumi, Mohamed Boussarsar

**Affiliations:** 1 Medical Intensive Care Unit, Farhat Hached University Hospital, Sousse, Tunisia; 2 Research Laboratory N° LR12SP09, Heart Failure, Faculty of Medicine of Sousse, University of Sousse, Sousse, Tunisia

Dear editor

We read with great interest the article of Duan et al. “Early prediction of noninvasive ventilation failure in COPD patients: derivation, internal validation, and external validation of a simple risk score” published in *Annals of Intensive Care* [1]. The authors debated the benefit of a simple risk-scoring system to predict noninvasive ventilation (NIV) failure, based on bedside clinical variables. Even though a meticulous approach was applied to establish the HACOR score with good predictive power for NIV failure in chronic obstructive pulmonary disease (COPD) patients, a few reservations could be discussed. As stated by the authors, the present study presented certain limitations, some of which could be the lack of generalizability due to disparities in quality of NIV and environmental conditions which were reflected in a lesser sensitivity and specificity when using the score in different hospitals. From our point of view, a few other observations could be raised:

First, the authors opted for a stratified scale in order to reach high predictive quality. However, multiple intervals for each clinical variable of the score could make its use complex and time-consuming. A risk score based on one cutoff for each element could be easier to recall and perform. We would like to ask whether the authors had tried to establish the predictive power of the score relying on only one cutoff for each variable before reaching the final version of the scale.

Second, the HACOR score was first calculated after 1–2 h from NIV initiation. The prediction of NIV failure was based on the score calculation on a given moment not taking into account the score variability from 1 h to the other. HACOR score variability between two time points could be more interesting to predict NIV failure than using a single HACOR calculation. In addition, modifying the score to focus on the evolution of the patients’ clinical state and biological parameters under NIV could be more compelling to detect late NIV failure [2]. In the 2017 article “Assessment of heart rate, acidosis, consciousness, oxygenation, and respiratory rate to predict noninvasive ventilation failure in hypoxemic patients” conducted by the same author, the difference between two time points in the HACOR score improved in hypoxemic patients with NIV success and remained unaltered in patients with NIV failure [3]. Identical reasoning could be applied for hypercapnic patients.

Establishing a score for NIV failure could be useful to detect the right moment to initiate mechanical ventilation with minimum risks and complications. Taking into consideration the early evolution under NIV of hypercapnic encephalopathy, ABG’s, simple bedside surrogates of patients’ respiratory mechanics and resulting ventilatory patterns could be of great predictive value.

## Data Availability

None.

## Abbreviations

NIV: Noninvasive ventilation
COPD: Chronic obstructive pulmonary disease
